# Ovarian Cancer Diagnosis and Chemoresistance Prediction Model Based on cfRNA Molecular Signature

**DOI:** 10.1002/advs.76274

**Published:** 2026-07-17

**Authors:** Qinhao Guo, Yangyang Zhang, Yongcheng Jin, Siwei Deng, Yongqi Chen, Zheng Feng, Hao Wen, Liu Wang, Yilin Li, Fanghong Ou, Yong Shen, Haiming Li, Tianyao Zhou, Xingzhu Ju, Xiaohua Wu

**Affiliations:** ^1^ Department of Gynecologic Oncology Fudan University Shanghai Cancer Center Fudan University Shanghai China; ^2^ Department of Oncology Shanghai Medical College Fudan University Shanghai China; ^3^ OxTium Technology Co., Ltd. Shenzhen Guangdong China; ^4^ Research Institute of Tsinghua University in Shenzhen Shenzhen Guangdong China; ^5^ Department of Biosciences and Bioinformatics School of Science Xi'an Jiaotong‐Liverpool University Suzhou Jiangsu China; ^6^ Department of Radiology Fudan University Shanghai Cancer Center Fudan University Shanghai China

**Keywords:** AI model, cfRNA, chemoresistance, diagnosis, ovarian cancer

## Abstract

**Background**: Ovarian cancer (OVCA) is a common and highly aggressive gynecologic malignancy often diagnosed at advanced stages. Approximately 30% of patients develop platinum resistance, resulting in disease recurrence and progression. Currently, no diagnostic or chemoresistance prediction model based on plasma cell‐free RNA (cfRNA) profiling exists.

**Methods**: We recruited 304 participants and performed plasma cfRNA sequencing. After quality control, 172 OVCA patients and 70 healthy controls were assigned to the training set, and 44 OVCA patients with 18 controls to the test set. A DenseNet‐based deep learning model was developed to analyze cfRNA features.

**Results**: The model distinguished OVCA patients from healthy controls with AUCs of 0.9997 in the training set and 0.9747 (95% CI: 0.9437–0.9963) in the test set. For chemoresistance prediction, it yielded AUCs of 0.9442 and 0.8421 (95% CI: 0.7504–0.9147), respectively. Our model outperformed comparative models across both tasks, though the performance advantage in the chemoresistance prediction task should be interpreted cautiously given the limited sample size. Interpretability analyses combined with bioinformatics identified FLOT1, IFITM3, and IFITM2 as putative diagnostic cfRNA biomarkers.

**Conclusion**: This plasma cfRNA‐based deep learning model identifies OVCA patients and predicts chemoresistance, offering a non‐invasive tool for early diagnosis and treatment stratification.

**Trial Registration**: This study was registered in the Chinese Clinical Trial Registry under registration number ChiCTR2500099940.

## Graphical abstract

A deep learning model analyzes cfRNA profiles extracted from the blood of OVCA patients. This innovative approach distinguishes OVCA from healthy controls with high accuracy. Crucially, it reliably predicts patient response to chemotherapy (sensitive versus resistant subgroups). This robust, AI‐powered strategy provides a non‐invasive tool for OVCA management and personalized treatment decision‐making.

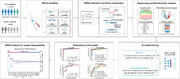

## 1 Introduction

Ovarian Cancer (OVCA) is a common and lethal gynecological disease. Globally, approximately 140 000 women die from ovarian cancer each year [1]. It often presents asymptomatically in early stages, which limits effective screening and diagnosis. Over 70% of cases are diagnosed at advanced stages, after the tumor has spread to other pelvic organs. Such advanced diagnosis reduces cure rates and worsens prognosis. Early diagnosis yields a 90% cure rate when the tumor is confined to one ovary [2].

Platinum‐based and taxane‐based chemotherapy are standard treatments for ovarian cancer. High‐dose chemotherapy can lead to complications and drug resistance, which diminish drug efficacy over time [3]. According to clinical criteria, patients with the disease are classified as chemotherapy‐resistant if disease recurrence or progression occurs within six months after initial platinum‐based therapy. Studies report that 20% to 30% of patients with OVCA show intrinsic resistance to chemotherapy. Early detection of chemotherapy resistance is crucial, as continued treatment without efficacy may accelerate disease progression in some cases [4, 5]. Despite this urgent need, effective clinical methods to reliably predict or identify chemotherapy resistance remain lacking, limiting the ability to tailor timely alternative therapies and improve patient outcomes.

Cell‐free RNA (cfRNA) detection offers distinct advantages for early cancer diagnosis and the identification of chemoresistance. cfRNA testing is a non‐invasive technique that collects bodily fluids such as blood, urine, or saliva to capture dynamic gene expression profiles, reflecting real‐time physiological and pathological states [6]. Unlike cell‐free DNA (cfDNA) that provides static genomic data, cfRNA encompasses a diverse range of RNA types, including mRNA, microRNA (miRNA), long non‐coding RNA (lncRNA), and circular RNA (circRNA), thereby enabling a broader and more dynamic molecular snapshot for earlier detection of cancers and emerging drug resistance [6]. Concurrently, advances in artificial intelligence (AI) analysis have facilitated the construction of high‐performance predictive models, providing more possibilities for optimizing the early detection of OVCA and drug resistance, and helping to identify key biomarkers [7].

This study used an observational retrospective cohort. It integrates high‐dimensional cfRNA data with bioinformatics and deep learning approaches. This established predictive models for disease progression and drug sensitivity in OVCA. Our primary objective is to identify reliable biomarkers that enable the early detection of OVCA and chemotherapy‐resistant patients, thereby facilitating timely clinical interventions to slow or prevent disease progression and improve patient outcomes.

## 2 Results

### 2.1 Study Design and Cohort Characteristics

**FIGURE 1:**
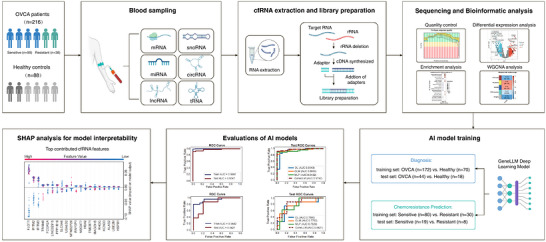
Study Overview. This study began with blood collection from a cohort of 216 ovarian cancer patients and 88 healthy controls, followed by plasma separation, cfRNA extraction, and cDNA library construction. Subsequent sequencing and bioinformatics analysis culminated in the development of a machine learning model for classifying ovarian cancer and predicting chemoresistance. The cohort was divided into training, test, and validation sets for robust AI analysis. Model performance was assessed using ROC curves, and interpretability was achieved through SHAP analysis. Abbr: OVCA, ovarian cancer; mRNA, messenger RNA; snoRNA, small nucleolar RNA; miRNA, microRNA; circRNA, circular RNA; lncRNA, long non‐coding RNA; tRNA, transfer RNA; rRNA, ribosomal RNA; cDNA, complementary DNA; cfRNA, cell‐free RNA; ROC, receiver operating characteristic; AUC, area under the curve; SHAP, SHapley additive exPlanations.

This study recruited participants from the Fudan University Shanghai Cancer Centre, comprising 216 histologically or cytologically confirmed ovarian cancer patients and 88 healthy controls. Plasma samples from all study subjects were processed using an established protocol for cfRNA extraction, library preparation, and sequencing (Figure 1). Quality control criteria included duplication rates below 60%, mapping rates above 60%, and mitochondrial ribosomal RNA (mt‐rRNA) content below 30% (Table S1)

Before analysis, subjects were divided into a training cohort (172 patients with OVCA and 70 healthy controls) for model development and hyperparameter tuning, and an independent test cohort (44 patients with OVCA and 18 healthy controls) was reserved for final evaluation without involvement in training or model selection (Table 1). The average ages of subjects with and without OVCA ranged from 57.04 to 59.91 in the training and test cohorts. In the training cohort, the proportions of chemosensitive and chemoresistant patients were 46.51% and 17.44%, respectively, while in the test cohorts, the corresponding proportions were 43.18% and 18.18% (Table 1).

**TABLE 1:** Patient characteristics of Training cohort and Testing cohort.

	Training Data Set		Test Data Set	
Patient Characteristic	Healthy (n = 70)	OVCA (n = 172)	Healthy (n = 18)	OVCA (n = 44)
Female, No.(%)	242 (100%)		62 (100%)	
Age (mean)	50.83	58.10	49.39	58.23
≤55	43 (61.43%)	63 (36.63%)	12 (66.67%)	16 (36.36%)
>55	27 (38.57%)	109 (63.37%)	6 (33.33%)	28 (63.64%)
CA125 at the first time (U/mL) (mean)	—	1855.88	—	1519.87
CA125 (U/mL)				
≤ 35	—	0 (0%)	—	1 (2.27%)
> 35	—	172 (100.00%)	—	43 (97.73%)
KELIM score (mean)	—	0.40	—	0.44
≥ 1	—	5 (2.91%)	—	2 (4.55%)
< 1	—	147 (85.47%)	—	30 (68.18%)
Lost	—	20 (11.63%)	—	12 (6.98%)
HE4 (pmol/L) (mean)	—	788.68	—	785.03
≤ 70	—	5 (2.91%)	—	1 (2.27%)
> 70	—	167 (97.09%)	—	43 (97.73%)
Recurrence or metastasis?				
Yes	—	67 (38.95%)	—	14 (31.82%)
No	—	105 (61.05%)	—	30 (68.18%)
Radiotherapy?				
Yes	—	1 (0.58%)	—	1 (2.27%)
No	—	171 (99.42%)	—	43 (97.73%)
FIGO stage				
I	—	5 (2.91%)	—	1 (2.27%)
II	—	7 (4.07%)	—	1 (2.27%)
III	—	97 (56.40%)	—	16 (36.26%)
IV	—	63 (36.63%)	—	26 (59.09%)
Chemotherapy Effect, No.(%)				
Chemosensitive	—	80 (46.51%)	—	19 (47.73%)
Chemoresistant	—	30 (17.44%)	—	8 (18.18%)
Lost	—	62 (36.05%)	—	17 (34.09%)

Abbr: OVCA, ovarian cancer; CA125, Cancer Antigen 125; KELIM score, CA‐125 ELIMination rate constant K score; HE4, Human Epididymis Protein 4

### 2.2 cfRNA Profiling in Ovarian Cancer

**FIGURE 2:**
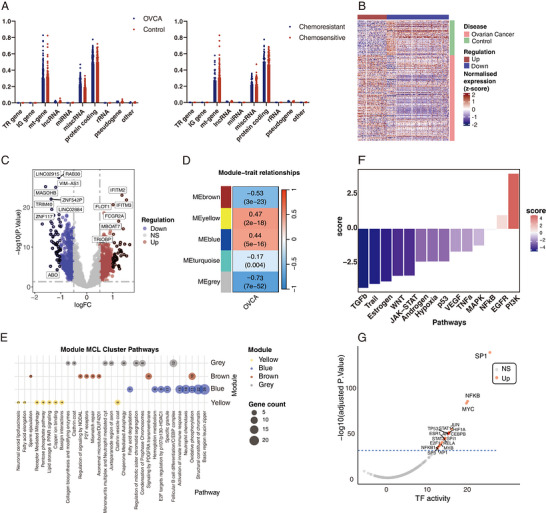
CfRNA Profiling Difference between patients with OVCA and healthy controls. (A) Distribution of RNA types in cfRNA samples comparing patients with OVCA versus healthy controls and chemoresistant versus chemosensitive patients with OVCA. The RNA biotypes were categorized based on Ensembl classifications with minor adjustments. The main categories included: Immunoglobulin (IG) gene, mitochondrial gene (mt‐gene, comprising mt‐rRNA and mt‐tRNA), T‐cell receptor (TR) gene, microRNA (miRNA), long non‐coding RNA (lncRNA), miscRNA, and ribosomal RNA (rRNA). A further ‘other RNA’ category was used for less frequent types, such as To be Experimentally Confirmed (TEC), small RNA (sRNA), small cytoplasmic RNA (scRNA), small Cajal body–specific RNA (scaRNA), small nuclear RNA (snRNA), small nucleolar RNA(snoRNA), vault_RNA, and artifact sequences. (B) The heatmap illustrates the differential expression profiling (FDR < 0.05 and |log_2_FC| > 0.5) between patients with OVCA (n = 216) and healthy controls (n = 88). (C) The volcano map illustrates the differential expression genes between patients with OVCA (n = 216) and healthy controls (n = 88). (D) Module‐traits heatmap from weighted gene co‐expression network analysis between OVCA phenotype and genes using weighted gene co‐expression network analysis. (E) Markov clustering analysis of the four modules most strongly correlated (|r| > 0.4, p < 0.05) with the OVCA phenotype (minimum STRING composite score = 0.4). (F) Pathway activity analysis comparing OVCA (n = 216) and healthy controls (n = 88). (G) Transcription factor (TF) activity profiles comparing OVCA (n = 216) and healthy controls (n = 88). Transcription factors with log(adjusted *p*‐values) higher than 30 are highlighted. (H) Abbr: OVCA, ovarian cancer.

Diverse RNA species, including protein‐coding mRNA, lncRNA, and miRNA were detected (Figure 1, Figure 2A, and Table S2). mRNA, mitochondrial RNA, and miscellaneous RNA, defined as any RNA product not characterized as other types, constituted the majority of cfRNA in both the patient and control groups (Table 2). The proportion of miscellaneous RNA was significantly increased in patients with OVCA (Figure 2A), indicating that various unexplored cfRNAs may be critical in cancer biology. Further deconvolution analysis revealed that this category was composed predominantly of 7SL RNA (∼96%) across all groups, whereas 7SK RNA and vault RNA were detected at negligible levels (Figure S1 and Table S14). Among Y RNA family members, RNY1 was the most abundant and showed higher abundance in OVCA samples, while RNY3 and RNY4 exhibited no significant differences between groups. Given the reported involvement of Y RNAs in DNA replication and cellular stress responses [8, 9, 10], the altered abundance of RNY1 suggests that it may represent an OVCA‐associated cfRNA signal.

**TABLE 2:** RNA type proportion.

(%)	protein coding	MiRNA	lncRNA	MiscRNA	TR gene	IG gene	mt‐gene	rRNA	pseudogene	other
OVCA	47.62	0.02	0.86	20.48	0.02	0.02	29.81	0.02	0.79	0.06
Control	49.87	0.03	1.00	16.97	0.02	0.02	31.22	0.01	0.79	0.07
Chemoresistant	50.39	0.02	0.78	21.70	0.02	0.02	27.21	0.01	0.80	0.04
Chemosensitive	46.96	0.02	0.80	19.76	0.02	0.02	30.44	0.02	0.81	0.06

Abbr: According to Ensembl Biotypes, RNA biotypes were categorized: IG gene (Immunoglobulin gene); mt‐gene (comprising mt‐rRNA and mt‐tRNA); TR gene (T‐cell receptor gene); miRNA (microRNA); miscRNA (miscellaneous RNA); rRNA (ribosomal RNA); lncRNA (long noncoding RNA); and other RNA. The ‘other RNA’ category consists of TEC (To be Experimentally Confirmed), ribozyme, sRNA (small RNA), scRNA (small cytoplasmic RNA), scaRNA (small Cajal body–specific RNA), snRNA (small nuclear RNA), snoRNA (small nucleolar RNA), vault_RNA, and artifact sequences.

Due to the subtle cancer‐associated signals and the inherently low abundance of cfRNAs, unsupervised analysis using principal component analysis (PCA) revealed no distinct separation between groups (Figure S2). To address this, we employed the limma‐voom method, which is specifically designed to detect small expression differences for RNA‐seq. This analysis identified 2,114 differentially expressed genes (DEGs) with a false discovery rate (FDR) < 0.05 and |log_2_FC| > 0.5 (Table S3). Heatmap visualization revealed distinct cfRNA expression patterns between patients with OVCA and healthy controls (Figure 2B). Volcano plot analysis highlighted several significantly upregulated genes in OVCA, including *IFITM2, IFITM3, FLOT1*, and *FCGR2A*, as well as downregulated genes such as *RAB30, MAGOHB*, and lncRNAs *LINC02915* and *VIM‐AS1* (Figure 2C). Further RT‐qPCR performed with previous cfRNA samples confirmed the significant upregulation of *FLOT1, IFITM2*, and *IFITM3* in OVCA (Figure S3A). Their dysregulation may influence ovarian cancer pathogenesis and represent potential diagnostic and therapeutic targets.

Weighted Gene Co‐expression Network Analysis (WGCNA) comparing OVCA and healthy cohorts identified five co‐expression modules (Figure 2D), with four demonstrating strong correlations with the OVCA phenotype (|r| > 0.4, *p* < 0.05). The yellow and blue modules exhibited significant positive correlations, whereas the brown and grey modules showed significant negative correlations with OVCA. Subsequent Markov cluster algorithm analysis (Figure 2E and Table S4) revealed distinct functional enrichments across these modules. The yellow module was enriched for mitophagy, pentose phosphate pathway, lipid storage, and PPAR signaling regulation, indicating metabolic reprogramming. The blue module included genes coding for structural constituents of chromatin and DNA‐binding proteins, indicating heightened TF‐chromatin interactions in OVCA. This module also showed enrichment in immune processes and metabolic pathways such as neutrophil chemotaxis, innate immune activation, uncoupling, fatty acid degradation, and oxidative phosphorylation (OXPHOS). OXPHOS pathway was also found in the negatively associated brown module, suggesting potential competing metabolic programs in OVCA [11]. Other pathways negatively associated with OVCA included PDGFRA transmembrane signaling and NODAL signaling in the brown module, as well as follicular B cell differentiation and regulation of mitotic sister chromatid segregation in the grey module. These results highlighted intricate biological processes underlying OVCA, primarily including metabolic changes, immune activity, signaling transduction, chromatin alterations, and cell cycle control.

Pathway activity analysis was performed using the decoupleR package with a Multiple Linear Model (MLM) method to estimate pathway activity scores based on coordinated expression changes of member genes [12]. Analysis revealed significant activation of PI3K (score = 4.01, corrected *p* < 0.05) alongside downregulation of TGFβ, TRAIL, Estrogen, WNT, JAK‐STAT, Androgen, Hypoxia and P53 pathways (score < 0, corrected *p* < 0.05) (Figure 2F and Table S5), suggesting substantial disruption of their regulatory functions in tumor microenvironment. Transcription factor (TF) analysis identified activated *SP1, NFκB, and MYC* in OVCA (score > 0, *p* < 0.05) (Figure 2G; Table S6). *SP1*, a key downstream target of the PI3K/AKT pathway, promotes tumor growth by increasing the expression of lipid metabolism genes such as PPAR pathway genes in the yellow module [13, 14] and regulating genes involved in cell division like mitotic regulation in the grey module, supporting proliferation and survival of cancer cells [15, 16].

Our analysis revealed cfRNA composition and expression profiles in OVCA. DEG analysis recovered several cancer‐related protein‐coding and non‐coding genes. Co‐expression modules and pathways highlighted disruptions in metabolism, immunity, signaling and cell cycle. Activation of critical transcription factors such as *SP1* underscores their contribution to OVCA progression through regulation of lipid metabolism and cell proliferation.

### 2.3 EV‐Associated Signatures in Ovarian Cancer

**FIGURE 3:**
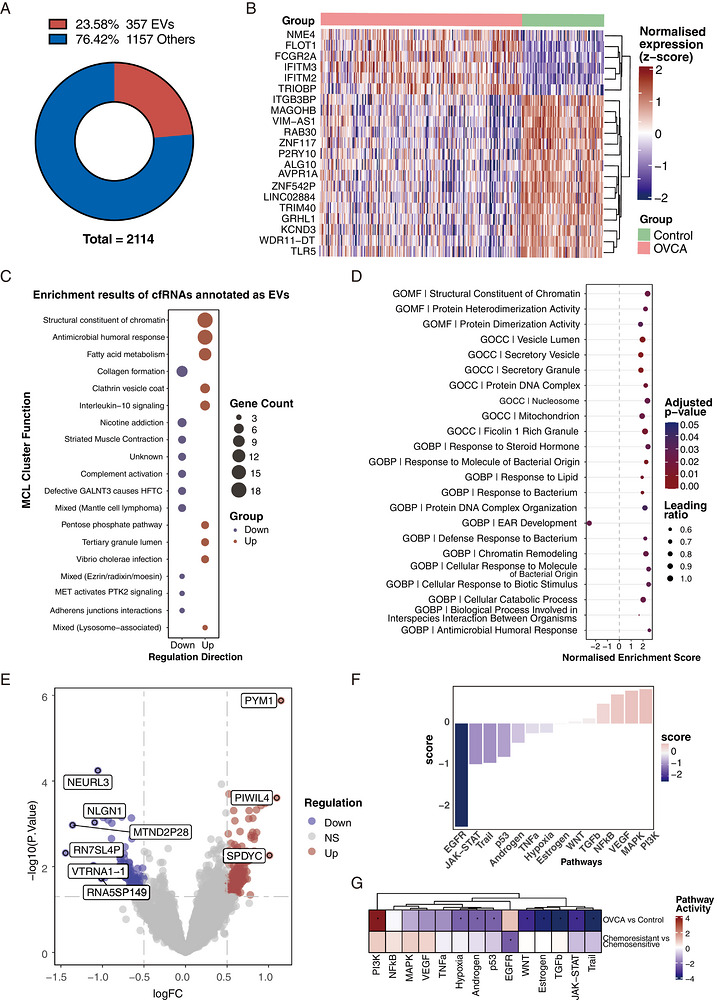
EV‐associated cfRNA Signatures in Ovarian Cancer and cfRNA Profiling Difference between chemoresistant and chemosensitive patients with OVCA. (A) Proportion of EV‐associated signatures in differentially expressed genes. At a threshold of FDR < 0.05 and |log_2_FC| > 0.5, 23.58% of significantly altered genes were identified as EV‐associated. (B) The heat map illustrates the differential expression profiling (FDR < 0.05, |logFC| > 1, LogFCSE < 0.15) between patients with OVCA (n = 216) versus healthy controls (n = 88). (C) Markov clustering analysis of EV‐associated signatures in differentially expressed genes filtered by FDR < 0.05 and |log_2_FC| > 0.5 and stratified by up/down‐regulation direction. (D) Gene set enrichment analysis of EV‐associated signatures (FDR < 0.01, 587 genes in total) based on Gene Ontology (including Biological Process (BP), Molecular Function (MF) and Cellular Component (CC) dataset), Top 10 significantly enriched terms from ranked by adj.p.val. (E) The volcano map illustrates the differential expression genes between chemoresistant (n = 38) and chemosensitive patients with OVCA (n = 99). (F) Pathway activity analysis comparing chemoresistant (n = 38) and chemosensitive patients with OVCA (n = 99). (G) Comparison heatmap of Pathway activity analysis in two groups (OVCA (n = 216) versus healthy controls (n = 88); chemoresistant (n = 38) versus chemosensitive (n = 99) patients with OVCA). Abbr: OVCA, Ovarian Cancer.

cfRNA studies indicated that a significant subset of these transcripts are associated with extracellular vesicles (EVs) [17]. EV‐associated signatures, actively released by cells, more accurately represent the gene expression profiles of their cells of origin, particularly aberrant tumor cells, making them suitable candidates for liquid biopsy [17]. We annotated differentially expressed cfRNAs using public EV databases (ExoCarta, Vesiclepedia, exRNA Atlas [18] and Table S7). At a threshold of FDR < 0.05 and |log_2_FC| > 0.5, 23.58% of significantly altered cfRNAs were identified as EV‐associated (207 upregulated, 150 downregulated) (Figure 3A). 21 cfRNAs met more stringent thresholds (FDR < 0.05, |logFC| > 1, LogFCSE < 0.15; Figure 3B), and five of them, including *FLOT1, IFITM3, IFITM2, TRIM40*, and *TRIOBP* were EV‐associated. These findings suggest that while many altered cfRNAs may result from passive release during apoptosis, a distinct EV‐associated subset may serve as biomarkers and play functional roles in OVCA pathology.

Further functional analysis via Markov clustering (Figure 3C, Table S8) revealed that upregulated EV‐enriched signatures based on database annotation were primarily enriched in pathways related to immune and inflammatory responses and cellular defence, such as antimicrobial humoral response, azurophil granule lumen, tertiary granule lumen, and interleukin‐10 signaling. This suggests active infiltration of immune cells like neutrophils in the tumor microenvironment. Additionally, upregulated cfRNAs were also involved in fatty acid metabolism and the pentose phosphate pathway, indicating metabolic adaptations supporting tumor growth and proliferation in OVCA. Conversely, downregulated cfRNAs were enriched in complement activation, potentially reflecting immune evasion mechanisms. Enrichments of gene sets involved in collagen formation and adherens junctions interactions (Figure 3C) suggested disruption in extracellular matrix remodelling and epithelial‐mesenchymal transition, which is consistent with an enhanced invasion and metastasis potential in OVCA.

GSEA enrichment analysis of the EV‐associated signatures using the Gene Ontology (GO) database (Biological Process, Molecular Function, Cellular Component) (Figure 3D), highlighted significant enrichment in pathways related to exosomal biogenesis and secretion, including Secretory Vesicle, Secretory Granule, and Vesicle Lumen, further validated the origin of these cfRNAs (FDR< 0.05). Pathways associated with gene expression and epigenetic regulation, and immune and inflammatory responses, were enriched (NES > 2). For instance, the enrichment of Ficolin‐1 Rich Granule (NES = 2.17, size = 25) underscored the presence of active immune cells. Enrichment was observed in pathways involved in cellular energy metabolism and hormone signaling, suggesting the potential occurrence of metabolic reprogramming in OVCA.

EV‐associated signatures not only serve as non‐invasive biomarkers reflecting tumor‐specific alterations and immune microenvironment activity, but also unveil key pathogenic processes including immune modulation, metabolic reprogramming, and facilitation of invasion in ovarian cancer.

### 2.4 Construction of a cfRNA‐Based Diagnostic Model for OVCA

**FIGURE 4:**
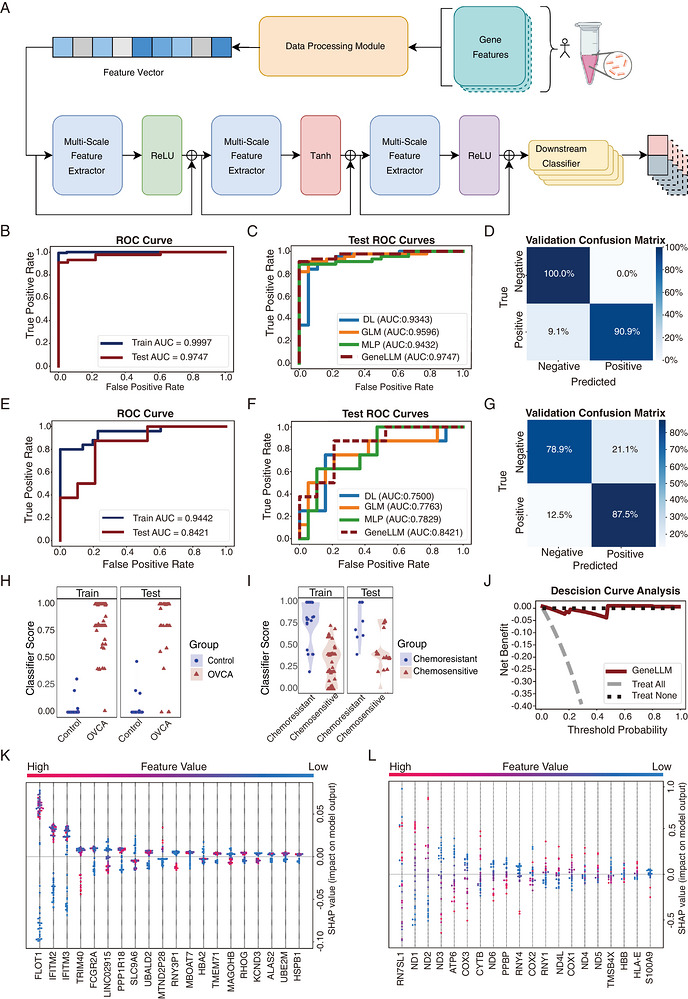
Model Building, Validation and Evaluation, and Interpretability Analysis. (A) Model architecture diagram based on cfRNA data. First, millions of cfRNA reads are processed through a data preprocessing module that includes bioinformatics analysis and normalization. Subsequently, the feature vectors are fed into a multi‐scale feature extraction module, which consists of multiple stacked multi‐scale feature extractors. Each extractor incorporates feed‐forward neural networks (FFNs) and skip connections, enabling the capture of multi‐scale patterns and variation characteristics in cfRNA sequences at different granularities. Finally, the integrated multi‐scale features are passed into a single feed‐forward classifier to produce a predictive probability indicating whether a patient has ovarian cancer. (B) ROC curves and AUC values of the GeneLLM framework for OVCA risk prediction in both the training cohort (n = 242, including 70 healthy controls and 172 OVCA) and the independent testing cohort (n = 62, including 18 healthy controls and 44 OVCA).(C) Comparative ROC analysis with corresponding AUC values between GeneLLM‐based OVCA risk prediction model and conventional models. (D) Confusion matrix of the GeneLLM‐based OVCA risk prediction model. (E) ROC curves and AUC values of GeneLLM framework for platinum‐based chemotherapy sensitivity/resistance prediction in training (n = 110, including 80 chemotherapy sensitivity and 30 chemotherapy resistance) and independent validation cohorts (n = 27, including 19 chemotherapy sensitivity and 8 chemotherapy resistance). (F) Comparative ROC analysis with corresponding AUC values between GeneLLM‐based chemotherapy response prediction model versus conventional models. (G) Confusion matrix for the GeneLLM‐based platinum chemotherapy sensitivity/resistance prediction model in patients with OVCA. (H) Classifier scores generated by the GeneLLM‐based OVCA risk prediction model. (I) Classification scores generated by the GeneLLM‐based platinum chemotherapy response prediction model for patients with OVCA. (J) Decision curve analysis using the GeneLLM‐based OVCA risk assessment model in a population with an OVCA prevalence of 1.1% (data from the Surveillance, Epidemiology, and End Results Program). (K) SHapley Additive exPlanations analysis results (Top20) of the GeneLLM‐based OVCA risk prediction model. (L) SHapley Additive exPlanations analysis results (Top20) of the GeneLLM‐based platinum chemotherapy response prediction model for patients with OVCA. Abbr: OVCA, ovarian cancer; AUC, Area Under the Curve; avg, average; CI, Confidence Interval; std, standard deviation; DL, deep learning; GLM, Generalized Linear Model; MLP, Multilayer Perceptron.

The preceding analyses established cfRNA expression differences between patients with OVCA and healthy controls. To explore the feasibility of constructing a cfRNA‐based diagnostic model for OVCA, we developed a deep‐learning model based on the GeneLLM architecture [19] (Figure 4A). The dataset was randomly split into training and validation sets at a 7:1 ratio, ensuring that the validation set was not used during model training to prevent data leakage. In the testing cohort, our model achieved an area under the curve (AUC) of 0.975 (95% CI: 0.9437–0.9963) (Figure 4B, Table 3), outperforming other traditional models, including Generalised Linear Models (GLM; AUC = 0.96), Multi‐Layer Perceptrons (MLP; AUC = 0.94), and Deep Learning (DL; AUC = 0.93) (Figure 4C). We further compared our model with four classical machine learning classifiers. Among the baseline models, XGBoost achieved the highest AUC (AUC = 0.968), followed by Logistic Regression and Random Forest (both AUC = 0.967), while Linear SVM showed the lowest value (AUC = 0.933) (Figure S4D, Table S15). Our proposed model achieved slightly higher performance (AUC = 0.975) than all baseline classifiers, demonstrating competitive or improved performance compared with conventional machine learning approaches. Additionally, the model demonstrated a sensitivity of 90.91%, specificity of 100%, and an F1 score of 0.952 (Figure 4D).

**TABLE 3:** Model performance evaluation indicators in two task.

		Diagnosis task	Chemoresistance task
AUC	avg	0.9747	0.8421
	95%CI	[0.9437, 0.9963]	[0.7504, 0.9147]
	std	0.0131	0.0405
Accuracy	avg	0.9355	0.8148
	95%CI	[0.8900, 0.9800]	[0.7398, 0.8900]
	std	0.0234	0.0418
Sensitivity (Recall)	avg	0.9091	0.875
	95%CI	[0.8524, 0.9851]	[0.7428, 1.0000]
	std	0.0336	0.0634
Specificity	avg	1	0.7895
	95%CI	[0.9615, 1.0000]	[0.6857, 0.8889]
	std	0.0118	0.0573
Precision	avg	1	0.6364
	95%CI	[0.9839, 1.0000]	[0.4883, 0.7917]
	std	0.0052	0.0792
F1 Score	avg	0.9524	0.7368
	95%CI	[0.9197, 0.9865]	[0.6129, 0.8438]
	std	0.018	0.0595
Negative Predictive Value (NPV)	avg	0.8182	0.9375
	95%CI	[0.7059, 0.9584]	[0.8723, 1.0000]
	std	0.0644	0.0324
True Positive		40	7
False Positive		0	4
True Negative		18	15
False Negative		4	1

Abbr: AUC, Area Under the Curve; Avg, Average; CI, Confidence Interval; Std, Standard Deviation

During model training, all hyperparameter tuning and model optimization were conducted exclusively within the training cohort using five‐fold cross‐validation to minimize overfitting. An initial grid search identified candidate hyperparameter ranges, which were further refined through empirical expertise. Model performance was rigorously evaluated at each stage via five‐fold cross‐validation, ensuring a robust selection process based solely on training data without test set involvement. Performance metrics of competing models are summarised in Table 3, while classification scores for both training and testing sets are visualized in Figure 4H. Stage‐stratified analysis revealed that the model attained 100% accuracy for FIGO stages I, II, and III, and 88.24% for stage IV on the test set, underscoring its promise for early detection of OVCA. Nevertheless, the small sample sizes of stages I and II warrant cautious interpretation of these results.

We conducted a leave‐one‐out ablation study by removing each RNA category from the full model to evaluate its contribution. Removing protein‐coding RNA or lncRNA led to a substantial decrease in AUC (to 0.42 and 0.46, respectively), indicating that these categories are the most critical contributors to model performance. In contrast, removing miRNA or miscRNA resulted in a more modest reduction (AUC = 0.70 and 0.75) (Figure S4F, Table S16), suggesting that their contributions are either smaller or partially redundant with the remaining features. Overall, the magnitude of AUC decrease reflects the relative importance of each RNA category to the full model.

Further decision curve analysis indicated that, in a population with an OVCA prevalence of 1.1% (data from the Surveillance, Epidemiology, and End Results Program), applying this model for clinical decision‐making at a threshold above 50% could avoid approximately 2 unnecessary interventions per 100 individuals, compared to intervening on all patients. These promising results demonstrated the high diagnostic reliability of the cfRNA‐based model (Figure 4J).

To improve model interpretability, we performed SHapley Additive exPlanations (SHAP) analysis. SHAP values provide a rigorous and intuitively interpretable measure of feature importance by assigning each gene a value reflecting its average contribution to the model's output. The top 20 features are visualized in Figure 4K, and the top 100 are listed in Table S12. High‐ranking contributors included *FLOT1, IFITM2, IFITM3, TRIM40, FCGR2A, and LINC02915*. Most of these features were previously identified as DEGs between OVCA and control groups (Figures 2C and 3B). Importantly, the top four features (*FLOT1, IFITM2, IFITM3, TRIM40*) are EV‐associated cfRNAs, further emphasizing the pivotal role of EV‐associated signatures in OVCA pathophysiology.

Collectively, these results underscore the strong diagnostic reliability and potential clinical utility of our cfRNA‐based deep learning model.

### 2.5 cfRNA Profiling in Chemotherapy‐Resistant OVCA

Patients with OVCA were classified as chemotherapy‐resistant if they experienced recurrence or progression within six months of initial platinum‐based therapy. After excluding patients who were lost to follow‐up or were non‐compliant (62 of 172 in the training set and 17 of 44 in the testing set), a complete‐case analysis was performed to define the final analytical cohorts, which include 110 patients in the training set (80 sensitive, 30 resistant) and 27 patients in the testing set (19 sensitive, 8 resistant) for model development and validation (Table 1).

To formally assess the potential for selection bias, a sensitivity analysis was performed to define the missing data mechanism. No significant differences in baseline characteristics were observed between patients included in the analysis and those lost to follow‐up, in either the training or testing cohort (Table 4). In particular, the KELIM score did not differ significantly between the two groups (0.43 vs. 0.33, *p* = 0.07). The KELIM score is a kinetic parameter quantifying the early CA‐125 elimination rate during chemotherapy, with a value ≥1.0 indicating favorable chemo‐sensitivity and predicting a higher likelihood of complete tumor resection and improved survival. As no evidence of systematic differences was found, the missing data mechanism was considered plausibly Missing Completely at Random (MCAR), and a complete‐case analysis was chosen to prioritize data integrity and consistency for the primary objective of model development.

**TABLE 4:** Comparison of baseline characteristics between patients lost to follow‐up and those who completed follow‐up.

	Training data set			Testing data set		
Baseline Characteristic	Completed (n = 110)	Lost to FU (n = 62)	p‐value	Completed (n = 27)	Lost to FU (n = 17)	*p*‐value
Age	59.05	56.42	0.10	59.19	56.71	0.36
CA125 at the first time (U/mL)	1899.00	1780.00	0.66	1641.00	1328.00	0.47
KELIM score	0.43	0.33	0.07	0.45	0.42	0.80
HE4	820.60	732.10	0.27	786.50	782.60	0.98

Abbr: FU, Follow‐Up; CA125, Cancer Antigen 125; KELIM score, CA‐125 ELIMination rate constant K score; HE4, Human Epididymis Protein 4

Unsupervised analysis of plasma cfRNA expression in the chemotherapy‐resistant patients (Figure S2B) provided initial insights into molecular differences. Differential expression analysis using limma‐voom identified *PYM1*, *PIWIL4*, and *SPDYC* as significantly upregulated in the chemotherapy‐resistant group (LogFC > 1, FDR < 0.01) (Figure 3E, Table S9). *PYM1*, involved in nucleotide metabolism, may enhance DNA repair or replication processes [20], helping cancer cells survive under chemotherapy‐induced DNA damage. *PIWIL4*, known for maintaining genomic integrity in germline cells [21], may be associated with cancer stem cell properties and epigenetic regulation [22]. *SPDYC*, a regulator of cell cycle progression by activating cyclin‐dependent kinases [23], may allow resistant cancer cells to bypass chemotherapy‐induced cell cycle arrest and continue proliferating despite treatment stress. RT‐qPCR results verified a significant upregulation of *SPDYC* in chemoresistant samples (Figure S3B). Pathway analysis (Figure 3F, Tables S10 and S11) revealed notable suppression of the EGFR signaling pathway in chemoresistant patients relative to the chemosensitive group (score = −2.47, *p* value = 0.014)(Figure 3G), suggesting that chemosensitive OVCA tumors may rely more heavily on EGFR signaling. These observations highlighted potential adaptive alterations in DNA repair, cell cycle progression, cancer stem cells, and signaling pathways associated with resistance mechanisms in OVCA, warranting further investigation into their clinical implications.

### 2.6 cfRNA‐Based OVCA Chemoresistance Model Also Demonstrates Strong Performance

To explore the feasibility of building a cfRNA‐based model for identifying chemotherapy resistance in patients with OVCA, we adapted the GeneLLM deep learning framework for the chemoresistance classification task. The dataset was randomly split into training and validation sets at a 7:1 ratio, with the validation set withheld during training to avoid data leakage. In the testing cohort, the model achieved an AUC of 0.842 (95% CI: 0.7504–0.9147) (Figure 4E, Table 3). The confusion matrix (Figure 4G) indicated a sensitivity of 87.50%, specificity of 78.95%, and an F1 score of 0.737. Evaluations of competing models were based on the optimal threshold derived by maximizing the Youden index on the ROC curve (sensitivity + specificity–1) (Table 3). Our model outperformed other traditional models, including GLM (AUC = 0.78), MLP (AUC = 0.78), and DL (AUC = 0.75) (Figure 4F). We further compared our model with four classical machine learning classifiers (Figure S4E). Among the baseline models, XGBoost achieved the highest AUC (AUC = 0.711), followed by Random Forest (AUC = 0.678), while Logistic Regression and Linear SVM showed the lowest performance (both AUC = 0.559). Our proposed model achieved higher performance (AUC = 0.842) than all baseline classifiers, demonstrating improved discriminative ability compared with conventional machine learning approaches. Through an iterative process of grid search and manual refinement evaluated by five‐fold cross‐validation, all hyperparameter tuning was confined to the training set, ensuring model selection robustness and isolation from the test set. Classification scores for training and test set samples in this task are visualized in Figure 4I.

We further benchmarked our cfRNA‐based model against the clinically established serum biomarkers CA125 and HE4, which are widely used for diagnosis and recurrence monitoring in OVCA. ROC analyses on the same testing cohort revealed that CA125, HE4, and their combined panel (CA125 + HE4) all yielded limited discriminative capability for chemoresistance prediction, with test AUCs of 0.592, 0.516, and 0.487, respectively (Figure S4A–C). This observation is consistent with previous reports that a single pre‐treatment measurement of these markers has a limited ability to predict platinum response, and that their predictive value largely depends on dynamic kinetics derived from serial measurements across multiple chemotherapy cycles, such as HE4 clearance after the third cycle or the modeled CA‐125 elimination rate constant (KELIM) [24, 25]. In contrast, our cfRNA model achieved a substantially higher AUC of 0.842 using a single pre‐treatment plasma sample, demonstrating clear superiority over these conventional serum markers and highlighting its practical value for early, non‐invasive chemoresistance prediction.

Besides, we conducted an ablation study by removing each RNA category from the full model to evaluate its contribution. The results showed that excluding protein‐coding RNA led to the most substantial decrease in performance (AUC = 0.309), indicating that this feature group is the most critical contributor to model performance. Removing lncRNA, miRNA, or miscRNA also resulted in notable performance reduction (AUC = 0.520, 0.533, and 0.563, respectively), with all values approaching random‐level performance (Figure S4G, Table S16). These findings suggest that each RNA category provides complementary predictive information and that the integration of all four categories is essential for achieving optimal classification performance.

To enhance the interpretability of our model, SHAP analysis was conducted to quantify the contributions of various features within the chemoresistance model. The top 20 most influential genes, including *RN7SL1* and several mitochondrial *ND* genes, are illustrated in Figure 4L, with the top 100 features listed in Table S13. Cell‐free *RN7SL1* has been demonstrated to activate the inflammatory response through the RIG‐I pathway in cancer and myeloid cells [26]. Mitochondrial *ND* genes encode subunits of Complex I in the mitochondrial electron transport chain, supporting the critical roles of mitochondrial reprogramming in OVCA chemoresistance [11, 27] In summary, these features represent important markers potentially regulating platinum‐based chemotherapy resistance in OVCA, offering valuable insights for biomarker development and therapeutic targeting.

## 3 Discussion

In this study, we performed cfRNA sequencing using plasma samples from 216 patients with OVCA and 88 healthy controls, and subsequently developed the first cfRNA sequencing data‐driven deep learning model for OVCA (Figure 1), which demonstrated strong performance in two distinct classification tasks: OVCA diagnosis (AUC = 0.975) and screening of platinum‐based chemotherapy resistance patients with OVCA (AUC = 0.842).

Initial bioinformatic analysis revealed 2,114 DEGs between patients with OVCA and healthy controls, including both protein‐coding mRNAs and various non‐coding RNA species (Figure 2A). The most significantly upregulated genes were EV‐associated *IFITM2*, *IFITM3*, and *FLOT1*, while *RAB30*, *MAGOHB*, and lncRNAs *LINC02915* and *VIM‐AS1* were notably downregulated (Figure 2C). *IFITM2* and *IFITM3* are members of the interferon‐induced transmembrane protein family, which have been implicated in cancer progression, including OVCA [28]. *FLOT1* encodes Flotillin‐1, a protein associated with membrane microdomains that facilitates cancer cell migration and metastasis. It has been correlated with OVCA progression and poor clinical outcomes [29]. Among the downregulated non‐coding genes, while there is limited information about *LINC02915, VIM‐AS1* has been reported to act as a tumor suppressor by promoting apoptosis in lung adenocarcinoma, while it functions as an oncogene by enhancing proliferation, migration, invasion, and EMT in several other cancers, thereby demonstrating that its functional role is highly context‐dependent and varies by cancer type and tissue environment [30, 31, 32]. *RAB30* and *MAGOHB* have roles in vesicle trafficking and mRNA processing, respectively. Their reduced expression may affect cellular homeostasis and cancer cell viability [33, 34]. The dysregulation of these genes suggested a possible loss of tumor‐suppressive functions in OVCA.

Further WGCNA analysis between OVCA and healthy groups identified five co‐expression modules (Figure 2D,E), which exhibited the intricate metabolic changes, immune activity, chromatin alterations, and cell cycle control underlying OVCA pathology. Pathway activity analysis (Figure 2F) outlines a typical but complex signaling pathway regulation map in OVCA. The pro‐survival PI3K/AKT pathway is activated, while multiple pathways involved in cell differentiation, apoptosis, anti‐proliferation, and stress responses are systemically downregulated. TF analysis (Figure 2G) highlighted *SP1*, which is a key downstream target of the PI3K/AKT pathway and has been implicated in ovarian tumorigenesis [15]. Collectively, these consistency between our results and previous reports increased the credibility of our data and analysis.

EV‐associated signatures have exhibited significant potential as non‐invasive biomarkers for early diagnosis, prognosis, and therapeutic monitoring in various cancers, including OVCA [35]. We specifically characterized the EV‐associated cfRNA profile in OVCA. At a threshold of FDR < 0.05 and |log2FC| > 0.5, 23.58% of significantly altered genes were identified as EV‐associated, including the most significantly upregulated *FLOT1*, *IFITM3*, and *IFITM2* (Figure 3A). EV‐associated RNAs tend to show consistent differential expression, which supports their robustness as biomarkers for OVCA detection and monitoring. Further functional analysis and GSEA enrichment analysis results were also consistent with known cancer hallmarks [36]. These suggest that extracellular vesicles and EV‐associated cfRNA are key mediators of molecular signals in the tumor microenvironment and circulation, underscoring the potential of EV‐associated cfRNA, such as *FLOT1*, *IFITM3*, and *IFITM2*, as promising biomarkers for OVCA non‐invasive diagnosis.

Moreover, the upregulated *FLOT1*, *IFITM3*, and *IFITM2*, as well as the downregulated lncRNA LINC02915 were among the top features contributing to our cfRNA‐based OVCA diagnostic model as interpreted by SHAP analysis. The OVCA diagnostic model achieved outstanding performance metrics, including an AUC of 0.975, sensitivity of 90.91%, specificity of 100%, and an F1 score of 0.952, surpassing traditional models such as GLM, MLP, and DL models in the same task (Figure 4). When compared with previous models based on DNA sequencing and methylation data that achieved AUC values ranging from 0.86 to 0.98 [37, 38, 39], our cfRNA‐based model also demonstrates comparable performance, reflecting the added value of transcriptomic information in capturing dynamic tumor biology. Decision curve analysis further highlighted the model's promise in improving diagnostic accuracy and patient management in OVCA.

Chemoresistance has been a major clinical challenge in OVCA treatment that leads to treatment failure and disease recurrence. Early identification and accurate classification of drug‐resistant subtypes in patients with OVCA are crucial to overcoming the challenges. In our subsequent analysis, patients lost to follow‐up or non‐compliant were excluded. Sensitivity analysis confirmed no significant baseline differences between included and excluded patients, indicating a low risk of selection bias. From DEG analysis, *PYM1*, *PIWIL4*, and *SPDYC* were identified as significantly upregulated in the chemotherapy‐resistant patients. Their functional roles have been linked to DNA repair capacity, cell cycle progression, cancer stem cell involvement, and signaling pathways, all of which have been implicated in OVCA chemoresistance mechanisms [3, 40], strengthening the reliability of the results. However, further experimental work in external cohorts is required to elucidate their precise mechanism underlying the OVCA chemoresistance.

Continuously, a cfRNA‐based OVCA chemoresistance model was constructed, achieving an AUC of 0.842, which surpasses that of existing traditional models. The top‐ranking feature selected through SHAP analysis, *RN7SL1*, has been established in breast cancer to be packaged into exosomes following activation of NOTCH‐MYC pathway in fibroblasts. Upon transfer, unshielded RN7SL1 activates RIG‐I/STAT1 signaling and induces interferon‐stimulated gene (ISG) expression, promoting tumor growth, metastasis, and chemotherapy resistance [41], suggesting a plausible role in OVCA. Mitochondrial *ND* genes, which encode subunits of Complex I in the mitochondrial electron transport chain, along with mitochondrial genes *COX1‐3, ATP6*, and *CYTB*, were also among the top‐ranking features. This supports the critical roles of mitochondrial reprogramming in OVCA chemoresistance, as documented in earlier studies [11, 27]. Involvement of *RNY1* and *RNY4* highlighted the importance of DNA replication and RNA stability regulation [42] in chemo‐resistance in OVCA. While *TMSB4X* promotes tumor proliferation and migration and is considered a biomarker [43], its specific role in resistance requires elucidation. Conversely, *S100A9* overexpression correlates significantly with cisplatin resistance, and its downregulation can overcome resistance and inhibit proliferation/migration [44, 45]. Collectively, these results could be corresponded to established mechanisms of platinum resistance in OVCA, including dysregulated drug transport, metabolic reprogramming, and enhanced DNA damage repair [45], providing more detailed reference for further investigation.

The pathway activity analysis in our results revealed the trend of upregulation of the EGFR pathway in OVCA. This finding aligns with prior studies reporting significantly elevated EGFR levels in the serum of patients with OVCA, identified as an early event in OVCA development [46, 47]. Previous research also indicates synergistic effects when combining EGFR and PI3K inhibitors in OVCA. EGFR expression is positively correlated with sensitivity to EGFR inhibitors, but inversely associated with sensitivity to PI3K or mTOR inhibitors [48]. Notably, our results demonstrated a decrease in EGFR pathway activity in platinum‐resistant OVCA, potentially indicating altered bypass signaling or compensatory mechanisms in the chemoresistant patients, while chemosensitive tumors may rely more heavily on EGFR signaling. These observations suggest that changes in pathway activity may be associated with sensitivity to chemotherapeutic agents in OVCA, warranting further investigation into their roles as predictive biomarkers and therapeutic targets for chemosensitive patients with OVCA.

Regarding the limitations of our study, although bootstrapping was performed to assess the stability of model performance, the relatively small sample size in the chemoresistance prediction task and the single‐center retrospective design of this study may introduce spectrum and selection biases, thereby restricting the generalisability of the findings to broader populations and other clinical settings. Potential confounding effects, such as cancer stage and sample quality factors like hemolysis, were not fully controlled, which could have influenced the observed results. In addition, detailed information on surgical history and specific chemotherapy regimens was not systematically documented in our retrospective records, which may have introduced residual confounding. Technical constraints related to library preparation and sequence mapping, especially for small RNAs and circular RNAs, may have limited the detection sensitivity and comprehensiveness of transcriptomic profiling. Additionally, extracellular vesicle attribution was inferential based on databases lacking physical EV isolation, which may affect the specificity of EV‐associated cfRNA findings. Despite these limitations, to the best of our knowledge, this represents one of the first cfRNA studies in ovarian cancer, providing a foundation upon which future prospective, multi‐center investigations incorporating patients with benign ovarian tumors can further validate the model's specificity and clinical utility in real‐world differential diagnostic scenarios.

In summary, our cfRNA‐based deep learning model effectively identifies patients with OVCA and those resistant to platinum‐based chemotherapy, offering promising potential for early screening and timely clinical intervention. Future functional and longitudinal studies with larger, multi‐center validation cohorts are essential to validate and extend these findings. Integrating other omics data, such as genomics and proteomics with cfRNA analysis may provide a more comprehensive understanding of the mechanisms underlying OVCA disease progression and chemoresistance.

## 4 Methods

### 4.1 Ethical Approval and Experimental Design

This study recruited participants from two cohorts at Fudan University Shanghai Cancer Center from 2019 to 2023, comprising 216 ovarian cancer patients and 88 healthy controls. All participants provided informed consent prior to joining the study, which was reviewed and approved by the Ethics Review Committee of Fudan University Shanghai Cancer Center (No. 2108241‐25). Inclusion criteria for OVCA groups are: patients with histologically or cytologically confirmed ovarian cancer; patients who have not received neoadjuvant chemotherapy or other anticancer treatments prior to therapy; patients willing to receive chemotherapy; patients with a sufficient expected survival time. Exclusion criteria are: patients with other severe systemic diseases that affect chemotherapy tolerance and efficacy; pregnant or breastfeeding women; patients with severe mental illness or cognitive impairment who cannot cooperate with treatment; patients allergic to components of chemotherapy drugs; patients with other malignant tumors.

### 4.2 Plasma Preparation

Plasma was prepared using centrifugation. Briefly, 18 mL of fresh blood was collected in vacutainer tubes containing EDTA‐K2 as an anticoagulant (KS Medical, Zhejiang, China) under aseptic conditions. The collected blood samples underwent a specific centrifugation protocol: 30 s acceleration, 2 min at 2700 rpm, 4 min at 2400 rpm, 4 min at 2700 rpm, 3 min at 3000 rpm, and 36 s deceleration and stop. This process separated the blood into three layers, with the uppermost layer being plasma.

### 4.3 cfRNA Extraction and Purification

CfRNA from 200 µL plasma samples was extracted using the EasyPure Total RNA Kit (Transgen, Beijing, China). The total cfRNA was eluted and collected with 15 µL of nuclease‐free water. After extraction, the concentration of the isolated RNA was estimated using the 2100 Bioanalyzer and RNA 6000 Pico Kit (Agilent, California, United States) following the manufacturer's instructions.

### 4.4 RT‐qPCR

Plasma cfRNA samples previously profiled by NGS were selected for RT‐qPCR validation. RT‐qPCR was performed in technical triplicate for each biological replicate using the Hifair Advanced One‐Step RT‐qPCR SYBR Green Kit (Yeasen, 11175ES70) according to the manufacturer's instructions. The thermal cycling program consisted of reverse transcription at 50°C for 6 min, initial denaturation at 95°C for 5 min, followed by 40 cycles of 95°C for 15 s and 62°C for 30 s. ACTB was used as the endogenous control. Relative expression levels were calculated using the ΔCq method, where ΔCq = Cq _target_ − Cq _ACTB_; when applicable, fold changes between groups were calculated using the 2^−ΔΔCq^ method. Primer sequences are listed in Supplementary Table S17.

### 4.5 Library Preparation

rRNAs—including cytoplasmic 28S, 18S, 5.8S, 5S, and mitochondrial 16S, 12S—were removed from the eluted RNA using the Hieff NGS One‐Step rRNA Removal Kit (Yeason, Shanghai, China), retaining mRNAs and other non‐coding RNAs. cfRNA‐seq libraries were prepared using the Hieff NGS Ultima Dual‐mode RNA Library Prep Kit (Yeasen, Shanghai, China) from 7.5 µL of eluted cfRNA, following the provided instructions, with 19 cycles of PCR amplification. Samples were barcoded with the Hieff NGS RNA 384 CDI Primer for Illumina t – 96 × 2 T Set 1 (Yeasen, Shanghai, China). Each sample was then pooled to 2 nM and sequenced on Illumina's NovaSeq X Plus using the PE150 strategy to an average depth of approximately 14 million reads per sample. Positive and negative controls were also sequenced to assess potential contamination during RNA extraction and library preparation. Nuclease‐free water and human brain RNA (Takara, California, United States) were processed using the same protocol as the plasma samples.

### 4.6 Data Preprocessing

For each sample, raw sequencing reads were quality‐checked using FastQC v0.12.0 and trimmed with fastp v.0.23.3. The processed reads were then mapped to the human reference genome (GENCODE human release 43) using STAR v.2.7.10b with ENCODE standard options. Finally, the mapped reads were quantified using featureCounts from the subread package v.2.0.6, generating a counts matrix.

### 4.7 Differential Expression Analysis

Differential expression analysis was conducted using the edgeR package v4.2.1 in R. Initially, gene expression data were filtered to remove lowly expressed genes, retaining only those with at least one count per million in at least one sample. The filtered data were then normalized using the trimmed mean of M‐values (TMM) method. A design matrix was constructed to model the experimental factors, including any batch effects or continuous variables. The voom method was applied to transform the count data to log2‐counts per million (logCPM) and to estimate the mean‐variance relationship. Linear models for each gene were fitted using the limma package v3.60.4, followed by empirical Bayes moderation to obtain more stable estimates of gene‐wise variability. Differential expression was assessed using moderated t‐statistics, and p‐values were adjusted for multiple testing using the Benjamini‐Hochberg method to control the false discovery rate. Batch effects and other unwanted sources of variation were removed from the normalized expression data using the removeBatchEffect function from the limma package, while preserving the variation associated with the treatment conditions of interest. Genes were considered significantly differentially expressed if they had an adjusted *p*‐value of less than 0.05. The results were sorted by p‐value and separated into up‐regulated and down‐regulated gene lists based on their log‐fold change values.

### 4.8 Weighted Gene Co‐Expression Network Analysis (WGCNA)

WGCNA was performed to identify co‐expressed gene modules and their associations with phenotypic traits using the WGCNA R package (v1.73). Normalized gene expression data (log2‐transformed CPM) were filtered to remove low‐expression genes (mean expression > 1 in ≥ 90% of samples), and a soft‐thresholding power (β = 4) was selected to construct a scale‐free network (R^2^ > 0.85). A signed adjacency matrix was converted into a Topological Overlap Matrix (TOM), and hierarchical clustering with dynamic tree cutting (minModuleSize = 30) identified co‐expression modules. Module‐trait correlations were calculated using module eigengenes (MEs), with significant associations (|r| > 0.4, *p* < 0.05) further analyzed for hub genes (kWithin top 10%).

### 4.9 Markov Cluster (MCL) Algorithm Analysis

At first, all the cfRNAs from key modules or EV annotations were input and performed by integrating multi‐source interaction data from public databases (STRING v11.5, BioGRID 4.4, IntAct 2023) encompassing experimentally validated, literature‐curated, and computationally predicted interactions. Confidence interactions (minimum STRING composite score = 0.4) were retained after removing redundancies. Networks were constructed using proteins as nodes and interactions as undirected edges (weighted by confidence scores). Topological properties, including degree, betweenness/closeness centrality, network diameter, and average path length, were calculated to identify hub proteins (top 5% by degree/betweenness). STRING database does not include non‐coding RNAs and pseudogenes, but our dataset contains a substantial proportion of protein‐coding genes. Given that non‐coding genes can regulate protein‐coding genes, the analysis of protein‐coding genes remains warranted and may yield valuable regulatory insights. Functional modules were detected using the MCL Algorithm implemented in the STRING database, with an inflation parameter of 3 to control cluster granularity. All statistical thresholds incorporated false discovery rate (FDR) control to minimize type I errors.

### 4.10 Functional Enrichment Analysis

Enrichment analysis for GO Biological Processes (BP) was conducted using the fgsea package v1.16.0 in R. The analysis utilized a ranked list of genes based on the log‐fold changes derived from the differential expression analysis. The fgsea algorithm was employed to calculate enrichment scores for each gene set, with analytical parameters set to perform 10 000 permutations. Gene sets included in the analysis were restricted to those containing a minimum of 10 genes and a maximum of 500 genes. *p*‐values obtained from the fgsea output were adjusted for multiple testing using the Benjamini‐Hochberg method. Gene sets with an adjusted *p*‐value of less than 0.05 were considered significantly enriched.

### 4.11 Pathway Activity Analysis

Pathway activity analysis was conducted using the decoupleR package v2.10.0 in R. A pre‐defined gene regulatory network specific to the organism of study (i.e. human) was used as the PROGENy network [49]. Normalized gene expression data were utilized to infer pathway activities across all samples. This was achieved using the Multiple Linear Model (MLM) method implemented in decoupleR. The method estimates the activity of each pathway based on the expression levels of its member genes, as defined in the reference network. A minimum of five genes per pathway was required for the analysis.

### 4.12 Transcription Factor Activity Analysis

We utilized the decoupler package v1.6.0 in Python to explore transcription factor (TF) activities. Our study leveraged the CollecTRI gene regulatory network, which maps interactions between human TFs and their target genes. To infer TF activities, we employed the Unified Linear Model (ULM) method available in the decoupler package. This method estimates TF activities by analyzing expression changes in their target genes, guided by the CollecTRI network.

### 4.13 GeneLLM‐based Deep Learning Model

A deep learning model for screening of OVCA was developed using cfRNA sequencing data. Millions of cfRNA reads were processed through a preprocessing module including bioinformatics analysis process and normalization. Feature extraction was performed with a deep neural network inspired by DenseNet, consisting of stacked multi‐scale extractor modules. Each module contained multiple feed‐forward layers (128 hidden units) with skip connections to preserve cross‐layer information. ReLU or Tanh activations and Dropout (0.3) were applied for regularization. The aggregated feature representation was passed to a feed‐forward classifier to estimate the probability of ovarian cancer. Patients were classified as cancer or non‐cancer based on a predefined global threshold. To improve robustness against class imbalance, we incorporated an AdversarialTrainingClassifier as the top‐level classification module. This classifier embeds adversarial training into the learning pipeline, simultaneously optimizing the primary task (e.g., diagnosis/chemoresistance) while strengthening resistance to input perturbations for better generalization. The classifier employs a dual‐branch structure: the main branch learns the primary classification objective, while the auxiliary branch introduces perturbed samples to minimize adversarial loss. For the loss function, we used Focal Loss (with parameter α determined by class imbalance and γ set to 2) to mitigate bias caused by extreme class imbalance. For optimization, both the diagnosis and chemoresistance tasks were trained with a batch size of 32 using the Adam optimizer (learning rate = 1e‐4, weight decay = 5e‐3), with training epochs set to 100 and 200, respectively. The GeneLLM‐based model employs a partitioning of the training cohort (7:1 for training and validation) and an ensemble learning strategy. This ensemble constitutes multiple sub‐models, whose predictions are integrated through a weighted averaging scheme to yield the final output. Performance metrics on the testing cohort were reported with 95% confidence intervals estimated from 1000 bootstrap resamples.

### 4.14 Conventional Models and Evaluation

To enable performance comparisons with the above deep learning model, we additionally developed six benchmark models: (1) A Generalized Linear Model (GLM) utilizing statistical features of cfRNA expression (mean, standard deviation) as input, implemented as a multinomial logistic regression model fitted via maximum likelihood estimation to establish baseline performance under linear separability assumptions; (2) A Multilayer Perceptron (MLP) with three fully‐connected layers (200–100‐output neurons) employing ReLU activation and 0.2 Dropout for regularization, capable of learning nonlinear patterns while lacking long‐range dependency modeling; (3) A lightweight Transformer model retaining token‐level embedding and a 2‐layer self‐attention structure to evaluate Transformer architecture's applicability to cfRNA expression analysis with moderate computational requirements; (4) A Random Forest (RF) classifier consisting of 500 decision trees with balanced class weighting, unrestricted maximum tree depth, and a minimum leaf size of 1, providing a nonlinear ensemble‐tree baseline capable of capturing feature interactions; (5) A Support Vector Machine (SVM) classifier with a radial basis function kernel, standardized input features, probability calibration, C set to 1.0, gamma set to scale, and balanced class weighting, used to assess margin‐based nonlinear discrimination; and (6) An Extreme Gradient Boosting (XGBoost) classifier using task‐specific binary or multinomial objectives, histogram‐based tree construction, AUC‐based evaluation, 500 boosting estimators, maximum depth of 3, learning rate of 0.03, subsample ratio of 0.8, and colsample_bytree ratio of 0.8, serving as a regularized boosting‐based comparison model. For high‐dimensional cfRNA expression inputs, these machine‐learning models were further combined with univariate SelectKBest feature filtering based on ANOVA F‐statistics, with 128 retained features before model fitting.

All implementations used Python 3.10.14 with numpy (1.26.4) and pandas (2.2.2) for data processing, while leveraging PyTorch (2.3.1), scikit‐learn (1.5.1), scipy (1.14.0) and XGBoost for modeling. Models were trained on the training cohort, with traditional machine‐learning models optimized through 5‐fold cross‐validation. Performance was assessed using AUC, sensitivity and specificity metrics, with visualizations generated via matplotlib (3.9.2) to ensure clinically meaningful and comparable results.

### 4.15 Decision Curve Analysis (DCA)

This was performed to evaluate the clinical utility of predictive models by quantifying net benefit across clinically relevant threshold probabilities (pt) ranging from 0 to 1 (0.01 increments). For each model, net benefit was calculated as [(True Positives/Ncase)×*p*—(False Positives/Ncontrol)×(pt/(1−pt)) × (1−*p)*], where N is the cohort size, *p* is the true prevalence rate of OVCA, and compared against “treat‐all” and “treat‐none” strategies using the Python dca package (v0.0.8). Interventions Avoided per 100 [X] = (Net Benefit / pt) * 100. In a population with OVCA prevalence of 1.1% (according to SEER database), for a clinician using a decision threshold of [pt], employing the model to guide clinical decisions yields an avoidance of approximately [X] unnecessary interventions per 100 individuals compared to a treat‐all strategy.

### 4.16 SHapley Additive exPlanations (SHAP)

To enhance model interpretability, we applied the SHAP (v0.48.0) framework, which quantifies feature contributions based on Shapley value theory. Transcripts were first input, and 100 random training samples were extracted as the background set. We then used shap.DeepExplainer, suitable for deep learning models. SHAP values indicate whether a feature increases (positive) or decreases (negative) the predicted probability, with their sum equaling the difference between the model bias and the sample's prediction. After obtaining SHAP outputs, transcripts without symbols were filtered. Through this analysis, we identified key cfRNA features for ovarian cancer prediction, supporting biomarker discovery and interpretability.

## Author Contributions

Q.G., Y.Z., Y.J., and S.D. are co‐first authors who contributed equally to this work. They led the project conception, design, and subject recruitment. Y.C., Z.F., H.W., and L.W. were responsible for the processing of patient blood samples for cfRNA extraction and sequencing. Bioinformatic analysis and machine learning model development were performed by Y.J., S.D., Y.L., F.O., and Y.S. The study was supervised by H.L., T.Z., X.J., and X.W. The original draft was written by Q.G., Y.Z., Y.J., and S.D. with input from all co‐authors. All authors reviewed, edited, and approved the final manuscript.

## Funding

This research was funded by National Natural Science Foundation of China (Nos. 82272898, 82203723, and 82471932), the Three‐Year Action Plan to Promote Clinical Skills and Clinical Innovation Capacity of Municipal Hospitals by the Shanghai Shenkang Hospital Development Center (SHDC2020CR5003‐001, SHDC2025CCS006), the CSCO‐CYH Oncology Research Fund (Y‐Young2024‐0297), the Autonomous Region Key Research and Development Program (No. 2024B03037‐1) and Shenzhen Science and Technology Program (No. 20240724152335001).

## Code Availability

All data preprocessing and downstream analyses were performed using standard bioinformatics tools on a Linux CentOS 8‐based High‐Performance Computing (HPC) system, with R version 4.1.3, supplied by OxTium Technology Co. Ltd. The names, versions, and detailed usage of all tools are described in the Methods section. Unless otherwise stated, all tools were run with their default parameters.

## Ethics Statement

All participants in this study were fully informed of the research objectives, procedures, and potential risks, and written informed consent was obtained from each individual prior to their enrollment. The study protocol was reviewed and approved by the Ethics Review Committee of Fudan University Shanghai Cancer Center.

## Conflicts of Interest

The authors declare no conflicts ofinterest.

## Supporting information




**Supporting File 1**: advs76274‐sup‐0001‐SuppMat.docx.

**Supporting File 2**: advs76274‐sup‐0002‐TableS1‐S17.zip.

## Data Availability

The data that supports the findings of this study are available in the supplementary material of this article.
