# Supplementary material for: Ovarian Cancer Diagnosis and Chemoresistance Prediction Model Based on cfRNA Molecular Signature
Source: Adv Sci (Weinh). 2026 Jul 17;13(52):e76274. doi: 10.1002/advs.76274 (PMC13379254; doi:10.1002/advs.76274)
Supplement: Supplementary file 1 [file ADVS-13-e76274-s001.docx]

**Supporting information**

**Title: Ovarian Cancer Diagnosis and Chemoresistance Prediction Model Based on cfRNA Molecular Signature**

Qinhao Guo*^†1,2^, Yangyang Zhang^†1,2^, Yongcheng Jin^†4,5^, Siwei Deng^†4,5^, Yongqi Chen^1,2^, Zheng Feng^1,2^, Hao Wen^1,2^, Liu Wang^1,2^, Yilin Li^4,5^, Fanghong Ou^4^, Yong Shen^4,6^, Haiming Li*^2,3^, Tianyao Zhou*^4^, Xingzhu Ju*^1,2^, Xiaohua Wu*^1,2^

**Figure S1. Deconvolution of the Miscellaneous RNA category in plasma cfRNA.**

(A) Relative composition of small non-coding RNA constituents (7SK, 7SL, Vault RNA, RNY1, RNY3, RNY4) within the Miscellaneous RNA category in OVCA versus healthy controls.

(B) The same compositional analysis between chemosensitive and chemoresistant OVCA patients.

Bars represent mean ± SD as percentage of total MiscRNA reads; dots denote individual samples.

**Figure S2: (A)&(B)** Principal component analysis of cfRNA expression profiles comparing patients with OVCA (n=216) versus healthy controls (n=88) (A) and chemoresistant (n=38) versus chemosensitive (n=99)patients with OVCA (B). (C) Comparative analysis before and after TMM normalization. (D) MA Plot of OVCA (n=216) vs Control Samples (n=88). Genes with significantly upregulated expression in OVCA samples (FDR < 0.05 and log2 FC > 0.5) are highlighted in red, while significantly downregulated genes (FDR < 0.05 and log2 FC < -0.5) are shown in green. Non-significant genes are displayed in gray.

Abbr: OVCA, ovarian cancer

**Figure S3. RT-qPCR validation of top feature genes identified by SHAP analysis in an independent cohort.**

(A) Quantitative reverse transcription polymerase chain reaction results of feature genes. The relative expression levels of FLOT1, IFITM2 and IFITM3 are compared between patients with OVCA and healthy controls (healthy controls, n=3; OVCA, n=6).

(B) Quantitative reverse transcription polymerase chain reaction results of feature genes. The relative expression levels of SPDYC between the groups with OVCA-chemosensitive(n=3) and OVCA-chemoresistant (n=3).

Bars represent mean ± SD; dots denote individual samples. *p < 0.05, **p < 0.01, ***p < 0.001.

**Figure S4. Comparative performance of conventional biomarkers, machine learning classifiers, and RNA biotype contributions in OVCA diagnosis and chemoresistance prediction.**

(A–C) ROC curves of conventional serum biomarkers for distinguishing chemosensitive and chemoresistant patients with OVCA: CA125 alone (A), HE4 alone (B), and the combined CA125 + HE4 panel (C). Training (blue) and test (red) AUC values are shown.

(D, E) Test-set ROC curves comparing four conventional machine learning classifiers (Logistic Regression, Random Forest, Linear SVM, and XGBoost) trained on identical feature inputs, in the OVCA diagnosis task (D) and the chemoresistance prediction task (E).

(F, G) Leave-one-out ablation analysis evaluating the contribution of each RNA biotype (protein-coding RNA, lncRNA, miRNA, MiscRNA) to model performance. Each curve represents the model retrained after excluding the indicated RNA category from the full feature set, in the OVCA diagnosis task (F) and the chemoresistance prediction task (G).

**Table S1:** Quality control for cell-RNA sequencing.

**Table S2:** RNA types proportion of all the samples.

**Table S3:** Differential analysis result between OVCA (n=216) and healthy controls (n=88)

**Table S4:** Markov clustering analysis result of the four modules most strongly correlated with the OVCA phenotype (|r| > 0.4, p < 0.05) .

**Table S5:** Pathway activity analysis result comparing OVCA (n=216) and healthy controls (n=88).

**Table S6:** Transcription factor activity analysis result comparing OVCA (n=216) and healthy controls (n=88).

**Table S7:** EV annotation of differentially expressed cfRNA filtered by FDR <0.05 and |log2FC| > 0.5.

**Table S8:** Markov clustering analysis result of EV-derived cfRNAs (FDR < 0.05 and |log2FC| > 0.5).

**Table S9:** The top 10 results of gene set enrichment analysis of EV-derived cfRNAs (FDR < 0.01, |log2FC| > 0.5, 587 genes in total) were obtained based on the Gene Ontology (including Biological Process, Molecular Function, and Cellular Component) and Reactome datasets.

**Table S10:** Differential analysis result between chemoresistant (n=38) and chemosensitive (n=99) patients with OVCA.

**Table S11:** Pathway activity analysis result comparing chemoresistant (n=38) and chemosensitive (n=99) patients with OVCA.

**Table S12:** SHapley Additive exPlanations analysis Top 100 features result of the OVCA risk prediction model.

**Table S13:** SHapley Additive exPlanations analysis Top 100 features result of the platinum chemotherapy response predicton model.

**Table S14:** Deconvolution analysis of the Miscellaneous RNA category in plasma cfRNA.

**Table S15:** Comparative performance of the proposed model and machine learning classifiers in OVCA diagnosis and chemoresistance prediction.

**Table S16:** Ablation analysis of RNA categories across different classification models.

**Table S17:** Primer Sequence.
